# Retraction: Opa1 reduces hypoxia-induced cardiomyocyte death by improving mitochondrial quality control

**DOI:** 10.3389/fcell.2022.1014307

**Published:** 2022-08-17

**Authors:** Frontiers Editorial Office

Following publication, concerns were raised regarding the validity of the data in the article. The authors failed to provide the raw data or a satisfactory explanation during the investigation, which was conducted in accordance with Frontiers’ policies.

Given the concerns, and the lack of raw data, the editors no longer have confidence in the findings presented in the article.

The authors do not agree to this retraction.

This retraction was approved by the Chief Editors of Frontiers in Cell and Developmental Biology and the Chief Executive Editor of Frontiers.

